# Validity of a two-item physical activity questionnaire for assessing attainment of physical activity guidelines in youth

**DOI:** 10.1186/s12889-015-2418-6

**Published:** 2015-10-23

**Authors:** Michelle Hardie Murphy, David A. Rowe, Sarahjane Belton, Catherine B. Woods

**Affiliations:** School of Health and Human Performance, Dublin City University, Dublin 9, Ireland; School of Psychological Sciences and Health, University of Strathclyde, Glasgow, UK

**Keywords:** Physical activity, Validity, Youth, Adolescence, Epidemiology

## Abstract

**Background:**

As physical activity is important for health and well-being, it is essential to monitor population prevalence of physical activity. Surveillance is dependent on the use of valid and reliable measurement tools. The PACE+ questionnaire is used globally in youth and has acceptable reliability; however it has not been validated in a European sample. The purpose of this study is to validate this instrument in a sample of 10–18 year old Irish youth.

**Methods:**

Participants (*n* = 419, 45.7 % male) completed the PACE+ two-item questionnaire and were asked to wear an Actigraph accelerometer for eight consecutive days. Freedson cut-points were used to estimate moderate to vigorous physical activity from accelerometer counts. Analyses compared self-report and accelerometry data in participants with (1) ≥5 and (2) seven valid accelerometer days. Calculations were performed for the whole sample, and were stratified by sex and school level (primary; post-primary).

**Results:**

Spearman correlations between self-reported physical activity levels and accelerometry derived minutes of moderate-to-vigorous physical activity per day were small (*r* = 0.27; seven valid days) to moderate (*r* = 0.34; ≥5 valid days). Higher correlations were found in older participants (post-primary *r* = 0.39; primary *r* = 0.24) and females (*r* = 0.39; males *r* = 0.27) using ≥5 valid days. The agreement level was high (68–96 %). The accuracy of classifying those not meeting the guidelines (specificity) was moderate to high (59–100 %).

**Conclusions:**

The PACE+ self-report instrument has acceptable validity for assessing non-achievement of the adolescent physical activity recommendations. The validity is higher in females and increases with age. The continued use of the tool is recommended and will allow for comparability between studies, tracking of physical activity over time including trends in youth population prevalence.

## Background

The benefits of physical activity (PA) to health are widely documented [1]. Monitoring and surveillance of population prevalence are of paramount importance [2] and vital for the progression of PA and public health [3]. Due to the complexity and multifaceted nature of PA, measurement of this behaviour can be challenging [4]. Valid and reliable PA measurement is essential for establishing prevalence, including trends over time [5], and verifying if efforts to promote PA are having a positive influence [6]. Prevalence rates are dependent on the instrument used to measure it [7]. The process of selecting a suitable questionnaire is based on two fundamental criteria; validity and reliability of the instrument [8]. The use of one valid and reliable tool across many countries would provide consistency and comparability of findings [9]. Such an instrument would need to be simple and adept at assessing compliance with physical activity guidelines (PAGL) for health at a population level [10]. In youth aged 5–18 years, the PAGL stand at a minimum of 60 minutes of moderate-to-vigorous physical activity (MVPA) on every day of the week [1]. Although PAGL were developed using predominantly self-report data [11], due to the limitations of self-report measures in youth, validation of these measures with accelerometers is commonplace [6, 10, 12–16]. This combined approach to the assessment of PAGL compliance provides for informed discussion on the accuracy of prevalence estimates.

In epidemiology, self-report questionnaires are frequently used due to their low cost and convenience [17]. Ease of administration is a factor determining survey choice for large scale studies [18]. A brief two-item PA screening tool (PACE+) was designed for use in adolescents [12]. The tool was first developed for use in a primary care setting with the function of identifying individuals not meeting the PAGL and who, from a health perspective, could benefit from intervention. It assesses the number of days adolescents engage in a minimum of 60 minutes of MVPA and is used as an indicator of habitual PA. It does not aim to describe PA behaviour beyond this. This instrument is widely used across the globe. It is utilised by 43 countries in the WHO HBSC questionnaire [19], and by up to 94 countries in the Global School-based Student Health Study [20–22] and other studies [23–26].

Test-retest reliability has been conducted on the instrument. In a Finnish study, it was assessed alongside a vigorous PA item. It was found to have acceptable reliability with intraclass correlation coefficients (ICC) ranging from 0.6 to 0.8 [27]. In a Chinese study with a sample of 11 and 15 year olds, an ICC of 0.82 (95 % CI 0.74–0.88) was reported for the first item (MVPA in the last seven days) and an ICC of 0.74 (95 % CI 0.64–0.82) for the second item (MVPA in a usual week) [28]. More recently, the tool showed moderate reliability, in the same age groups, with an ICC ranging from 0.51 to 0.98 in three Eastern European countries. The ICC for the whole sample was 0.60 (95 % CI 0.55–0.64) [29]. No significant sex or age differences were noted in the Chinese or European studies described. These studies address the reliability of the PACE+ but not its validity.

Elsewhere it has been validated via correlations with accelerometer derived MVPA. It demonstrated moderate validity in a sample of 11–13 year old youth in the U.S. (*r* = 0.40, *p* < 0.001; test-retest ICC = 0.77) [12] and later in an Australian sample of 15–17 year olds (*r* = 0.40 with five days accelerometer data, *r* = 0.49 with seven days accelerometer data) [10].

Questionnaires appraised in one population cannot be systematically transferred to other geographical regions or populations [17]. There is a strong need for assessment of the validity of these items across the regions which utilise it [29]. Particularly as results from studies using this instrument are used for health promoting strategies and policies targeting youth [19]. It is also essential to validate it across all adolescent years. The need for further studies that investigate the validity of this instrument using objective PA monitoring has been highlighted in the literature [29]. The purpose of the study was to examine the validity of the PACE+ questionnaire for assessing physical activity, and attainment of the European physical activity recommendations of 60 minutes of MVPA per day through accelerometry.

## Methods

This study was conducted within the Children’s Sport Participation and Physical Activity (CSPPA) Study [23]. Initially, data were collected from a nationally representative sample (*N* = 5397) of youth from the Republic of Ireland using a systematic, one-stage cluster sampling method. A follow-up study took place five years after the original study. The validation study included a convenience sample of 419 participants (*n* = 284 from 2009; *n* = 135 from 2014) from 19 schools. Standardised procedures were adopted during data collection in 2009 and 2014 (both February – May). Dublin City University’s Research Ethics Committee approved the study in 2009 and 2014. Written informed consent was obtained from adolescents aged 18 years and written assent, in addition to, parental consent was gathered from participants <18 years.

Participants completed a self-report questionnaire, which included demographic information (sex, date of birth, nationality, area of residence and social class) as well as the PACE+ questions. Questionnaires were administered in primary (5^th^ and 6^th^ class; age 10–12 years) and post-primary (1^st^ to 6^th^ year; age 12–18 years) schools, from across the Republic of Ireland, in the presence of a research team member. Participants were provided with definitions of moderate and vigorous effort and instructed to only include activities of this intensity when completing the PACE+ questions. The first item asked them to report the number of days (0–7) they were physically active for at least 60 minutes per day in the past seven days. The second item asked the same question with respect to a typical or usual week [12]. An average value of the two items yielded a score of days per week that participants accumulated 60 minutes of MVPA. Compliance with PA recommendations was assessed in two ways; by creating binary variables for those achieving/not achieving ≥5 days MVPA and 7 days MVPA.

Additionally, PA was objectively measured using the ActiGraph accelerometer (GT1M and GT3X). This monitor is an acceptable criterion measure for evaluating questionnaire validity [30] and the most widely used for this purpose [17]. Accelerometers were distributed to participants on the same day as questionnaire completion. Participants were instructed to wear the devices for eight consecutive days on their right hip during all waking hours, except for when they were swimming or bathing. The eighth day was excluded from analysis as accelerometers were collected during the daytime. The epoch length was set at 15 s. Data from the devices were downloaded and cleaned using ActiLife software. Consecutive zero counts of 20 minutes or more were eliminated from total wear time.

Accelerometer data were analysed using Freedson age-specific cut points [31, 32] which have demonstrated excellent specificity and sensitivity for MVPA [33]. Similar to the NHANES study [34] and the validation study by Ridgers and colleagues [10], a threshold of four METs for moderate activity was chosen. A summary score of counts per min (CPM) represented total PA. For comparability purposes, compliance with PA recommendations was established using the same two methods as Ridgers and colleagues [10]; the average method and the all days method. In the average method (AM), the average minutes of MVPA per valid days was calculated and dichotomised as above or below 60 mins/day. In the all days method (ADM), valid days that participants engaged in ≥60 min of MVPA was determined and dichotomised into those meeting/not meeting (a) ≥ 5 days and (b) 7 days.

### Statistical analysis

Descriptive statistics were calculated for demographic, self-report and accelerometer data. For inclusion in the study, participants were required to have complete self-report data and meet accelerometer wear time criteria of a minimum of eight hours per day on at least five days. The sample that met inclusion criteria was compared to the full sample for sex, age, school level and self-reported days of ≥60 minutes of MVPA. All statistical analyses were performed for the whole sample and stratified by sex and school level. All calculations were completed using those with (a) ≥5 valid accelerometer days (*n* = 235; 41.3 % male) and (b) a subsample with seven valid accelerometer days (*n* = 77; 36.4 % male). Spearman rho correlation coefficients were calculated between self-report (average days 60mins MVPA) and accelerometry data (mins of MVPA/day; CPM). The strength of the correlations were ranked as small (>0.1), moderate (>0.3) and strong (>0.5) [35]. The percent agreement between self-report and accelerometry was established by assessing the consistency of the classification of achieving the PAGL between the two methods. Sensitivity was defined as the accuracy of classifying those achieving the PAGL. Specificity referred to the accuracy of classifying those who did not meet the PAGL. The positive predictive value (PPV) is the percentage who self-reported meeting PAGL who actually met the guidelines and the negative predictive value (NPV) is the percentage who self-reported not meeting PAGL who did not actually meet the guidelines [36]. All analyses were performed using SPSS v.21 (IBM Corp, Armonk, NY).

## Results

Of the 419 youth (45.7 % male) aged 10–18 years who participated in the study, 56.1 % (*n* = 235; 41.3 % male; 14.7 ± 3.1 years) met the inclusion criteria. Participants were excluded from the analysis if they were missing one item of the screening tool (*n* = 6) or if they did not meet accelerometer wear time criteria (*n* = 175). A malfunction in accelerometer files led to the loss of three participants. Participants attended both primary (37 %; 11.2 ± 0.4 years) and post-primary (63 %; 16.8 ± 1.9 years) school levels. Table 1 presents the participant characteristics.

**Table 1:** Descriptive characteristics of study participants

	Full sample	Included sample		
		Primary	Post-primary	Total
	*n* = 419	*n* = 87	*n* = 148	*n* = 235
Age (mean ± SD)	14.8 ± 3.1	11.2 ± 0.4	16.8 ± 1.9	14.7 ± 3.1
Age (range)	10 - 18	10 - 12	12 - 18	10 - 18
Sex (%)				
Male	45.7	32.2	46.6	41.3
Female	54.3	67.8	53.4	58.7
Nationality (%)				
Irish	93.8	93.1	91.9	92.3
Other	6.2	6.9	8.1	7.7
Area of residence (%)				
Urban	43.4	57.6	41.5	47.4
Rural	56.6	42.4	58.5	52.6
Social class (%)				
Low	9.4	9.3	9.3	9.2
Medium	37.7	41.9	36.9	38.9
High	47.6	40.7	50	46.3
Unknown	5.3	8.1	3.8	5.6

There were no significant differences between the final sample and those excluded in terms of age (t(414) = 0.84, *p* = 0.40), school level (*X*^*2*^(1) = 1.38, *p* = 0.14) or self-reported days of 60 min MVPA (t(407) = 0.16, *p* = 0.87). However, a difference in sex was found (*p* = 0.03) with girls more likely to comply with wearing the accelerometer than boys (61.1 vs. 51.1 %).

Table 2 shows PA levels and compliance with PAGL for both self-report and accelerometer data. Across participant groups, the proportion meeting PAGL on all days of the week was low in both self-report (4–8 %) and accelerometry (AM 12–25 %; ADM 0–2 %). Rates were higher for meeting PAGL on ≥5 days per week (self-report 30–45 %; AM 12–32 %; ADM 6–16 %). Males had significantly (*p* < 0.01) higher values than females for MVPA mins/day, CPM and self-reported days of PA for ≥5 valid accelerometer days but not for seven days of data. Primary level students scored higher than post-primary students (*p* < 0.01) for MVPA mins/day (≥5 and 7 days) and total PA (≥5 days only).

**Table 2:** Physical activity levels and proportion achieving physical activity recommendations assessed by self-report and accelerometry

	Total	Males	Females	Primary	Post-Primary
≥5 valid accelerometer days	*n* = 235	*n* = 97	*n* = 138	*n* = 87	*n* = 148
Physical activity levels (mean ± SD)					
MVPA (mins/day)	40.8 ± 28.7	47.2 ± 34.5	36.3 ± 22.9	57.9 ± 24.1	30.8 ± 26.5
Total PA (CPM)^a^	238.5 ± 111.7	267.0 ± 123.2	218.5 ± 98.4	271.1 ± 86.9	219.4 ± 120.2
Self-reported PA (days)^b^	4.0 ± 1.7	4.6 ± 1.5	3.7 ± 1.8	4.3 ± 1.4	3.9 ± 1.9
Meeting 5 day PA recommendations (%)					
Self-reported PA ≥ 5 days^c^	36.6	45.3	29.7	42.4	32.5
Accelerometer (average method)^c^	19.6	30.9	11.6	32.2	12.2
Accelerometer (all days method)^d^	9.8	15.5	5.8	14.9	6.8
Seven valid accelerometer days	*n* = 77	*n* = 28	*n* = 49	*n* = 28	*n* = 49
Physical activity levels (mean ± SD)					
MVPA (mins/day)	38.7 ± 26.2	41.7 ± 30.2	37.0 ± 23.8	51.7 ± 16.4	31.3 ± 28.0
Total PA (CPM)^a^	223.5 ± 100.7	237.1 ± 106.9	215.7 ± 97.2	245.0 ± 58.8	211.1 ± 117.0
Self-reported PA (days)^b^	3.9 ± 1.7	4.2 ± 1.6	3.7 ± 1.8	4.2 ± 1.2	3.7 ± 1.9
Meeting 7 day PA recommendations (%)					
Self-reported PA = 7 days	5.2	7.1	4.1	3.6	8.2
Accelerometer (average method)^c^	16.9	25.0	12.2	25	12.2
Accelerometer (all days method)^e^	1.3	0	2.0	0	2.0

^a^CPM = counts per minute

^b^Self-reported PA: composite score of the two self-report items for days per week achieving 60 min MVPA

^c^Average method (AM): proportion achieving an average of 60 min or more of MVPA across all valid days

^d^All days method (ADM ≥5 days): proportion achieving ≥ 60 min MVPA on at least 5 days

^e^All days method (ADM 7 days): proportion achieving ≥ 60 min MVPA on all 7 days

Correlation coefficients were small to moderate (*r* = 0.27–0.34) between self-reported days meeting 60 minutes of MVPA and accelerometer data in terms of minutes of MVPA per day and total PA per day in the whole sample (Table 3). Stronger correlations were found in older participants (post-primary, *r* = 0.36–0.39; primary, *r* = −0.12–0.25) and girls (*r* = 0.38–0.41; males, *r* = 0.10–0.27) using both ≥5 and seven days. Correlations were significant for girls, post-primary students and the total sample using seven accelerometer days and in all groups using ≥5 days.

**Table 3:** Spearman rho correlations between self-reported and accelerometry recorded physical activity levels

		Self-reported PA^a^	
	(n)	MVPA (mins/day)^b^	Total PA (CPM)^c^
≥5 valid accelerometer days			
Total	235	0.34**	0.33**
Sex			
Male	97	0.27**	0.25*
Female	138	0.39**	0.38**
School level			
Primary	87	0.24*	0.25*
Post primary	148	0.39**	0.36**
Seven valid accelerometer days			
Total	77	0.27*	0.30**
Sex			
Male	28	0.10	0.16
Female	49	0.40**	0.41**
School level			
Primary	28	−0.12	−0.10
Post primary	49	0.39**	0.39**

^a^Self-reported PA: days per week achieving 60 minutes of MVPA

^b^Accelerometer derived average minutes of MVPA per day (MVPA mins/day)

^c^Accelerometer counts per minute (CPM)

*p < 0.05

**p < 0.01

Details of agreement, sensitivity, positive predictive value (PPV), specificity and negative predictive value (NPV) between self-reported PA and accelerometer data are displayed in Table 4. There was a high level of agreement between the PACE+ and accelerometer data. Using the AM, the agreement level was 68–85 % for ≥5 valid days and 71–82 % for seven days. For the ADM, agreement was 89–91 % for ≥5 days and 88–96 % for seven days of accelerometer data. Overall, the accuracy of classifying those achieving the guidelines (sensitivity) was low to moderate (≥5 days; 38–67 % accuracy) and in some cases not computable due to a lack of participants meeting the PAGL (7 days; not computable to 17 % accuracy). Values were higher in males than females (AM 67 vs. 38 %; ADM 67 vs. 50 %). The percentage of male and primary students who self-reported meeting the PAGL, who actually met them (PPV; 9–50 %) was higher than in female and post-primary students. The accuracy of classifying those not meeting the guidelines (specificity) was moderate (≥5 days; 59–72 % accuracy) to high (7 days; 92–100 % accuracy). The NPV was high (74–100 %) across all analyses.

**Table 4:** Agreement, sensitivity and specificity between self-reported physical activity and accelerometer data for compliance with recommendations

	≥5 valid accelerometer days (*n* = 235)					Seven valid accelerometer days (*n* = 77)				
	Agreement	Sensitivity	PPV^c^	Specificity	NPV^d^	Agreement	Sensitivity	PPV^c^	Specificity	NPV^d^
	(%)	(%)	(%)	(%)	(%)	(%)	(%)	(%)	(%)	(%)
Average method^a^										
Total	78.7	56.5	30.6	68.8	86.7	77.9	7.7	25.0	95.3	83.6
Sex										
Male	72.2	66.7	45.5	64.2	81.1	75.0	14.3	50.0	95.2	76.9
Female	83.3	37.5	14.6	71.3	89.7	79.6	NC	NC	95.3	87.2
School level										
Primary	67.8	53.6	40.5	62.7	74.0	71.4	NC	NC	100	75.0
Post Primary	85.1	61.1	22.9	71.5	93.0	81.6	16.7	25.0	93.0	88.9
All days method^b^										
Total	90.2	60.9	16.5	66.5	94.0	90.2	NC	NC	93.4	98.6
Sex										
Male	88.7	66.7	22.7	58.5	90.6	92.9	NC	NC	92.9	100
Female	91.3	50.0	9.8	71.5	95.9	89.8	NC	NC	95.8	97.9
School level										
Primary	93.1	61.5	21.6	60.8	90.0	96.4	NC	NC	100	100
Post Primary	88.5	60.0	12.5	69.6	96.0	87.8	NC	NC	91.7	97.8

*NC* not computable as no participants met the PAGL using all 7 days method

^a^Average method (AM): proportion achieving an average of 60 mins or more of MVPA across all valid days

^b^All days method (ADM): proportion achieving ≥ 60 mins MVPA on (i) ≥5 days (ii) 7 days

^c^PPV: positive predictive value

^d^NPV: negative predictive value

## Discussion

The purpose of this study was to examine the validity of a short questionnaire for assessing attainment and non-attainment of the youth PA recommendations among Irish youth.

Overall, the self-report questionnaire was moderately correlated with accelerometer data in terms of MVPA mins/day and CPM. The validity of the instrument was highest in girls and older adolescents. The low correlations in the younger group (primary) are consistent with findings in the literature [37]. A systematic review of PA questionnaires in youth revealed that adolescents’ self-report data correlated better with accelerometer scores than children’s [38]. This may be explained by their cognitive maturity and enhanced ability to recall PA with age [39].

The agreement level was high and varied across the different methods of analysis. As expected, the percent agreement was consistently higher using the ADM (≥88 %) than the AM (68–85 %). This illustrates a strong agreement between self-report and accelerometer data for detecting whether adolescents engage in the recommended levels of PA. Sensitivity results were low using the seven-days criteria, and in many cases it was non-computable. This can be attributed to the small proportions actually meeting the PAGL. Consequently, these results should be viewed with caution. Higher values in boys may be explained by the higher PA levels in boys than girls, and therefore, greater proportions meeting the PAGL. Similar trends were found for PPV. On the contrary, the accuracy of classifying those not meeting the PAGL (specificity) was moderate to high, and the NPV was consistently high. As specificity and sensitivity are inversely proportional [36], it is unsurprising that results for specificity are much higher. Nonetheless, it is important to identify this group for health promoting efforts.

To date, two studies aimed to validate this measurement tool, the first in a U.S. sample [12] and the second in a sample of Australian youth [10]. In the U.S. study [12], the overall correlations – based on PAGL on ≥5 days per week only - were greater than the current study (0.40 vs. 0.34). Similar ages between the post-primary students in the current study and the sample in the Australian study allow for direct comparison. Overall correlations were similar for MVPA mins/day (0.40 (Australia) vs. 0.39 (Ireland)) and for CPM (0.42 vs. 0.36) using ≥5 valid days. Correlations reported in both the U.S. and Australian papers can be described as moderate.

The overall agreement level was higher in the current study (78–90 %) than in the other two papers (63 % and 72–88 %). Higher sensitivity values in the Australian study are due to higher proportions meeting the PAGL. In the total sample, specificity was higher in this study compared with the Australian study over seven days but lower in the ≥5 days analysis. Regardless, specificity was good in both.

Furthermore, it is necessary to compare the PACE+ with other available self-report questionnaires. In the literature, Spearman rho correlations are the most commonly used measure of criterion validity for self-report instruments [17]. Review studies on PA questionnaires developed for use in children and adolescents found that the majority of instruments have acceptable reliability, and validity is low to moderate at best [40]. A systematic review found median validity correlations ranged from 0.22 to 0.41 [17]. All of these studies have a range of PA outcome measures including PA summary scores, total minutes of PA, MET minutes and MVPA minutes per day. Any of these measures can be used to categorise the respondent into meeting PAGL versus not meeting. A separate review of 89 PA measures for population surveillance in youth approved three study questionnaires, two of which contained the PACE+ [18]. In these studies, the PACE+ was used alongside other measures that describe PA behaviour.

The issue of overestimation of PA by subjective recall methods is frequently raised in the literature. A systematic review revealed that of those studied, 72 % of indirect PA measures overestimated objectively measured values [40]. In this study, the proportion achieving 60 minutes of MVPA on ≥5 days per week was higher in self-report than objective measurement. However, a reverse of this occurs when examining seven days. The averaging of two items to form the self-report score could potentially create a confounding effect by making it harder to achieve seven days.

In relation to PA levels, the self-reported levels described here are comparable to those found in a nationally representative sample of Irish students (*n* = 5397; aged 10-18years) from which these participants are extracted [23]. The mean days meeting 60 minutes of MVPA (4.0 ± 1.7 and 4.0 ± 1.8) were very similar. Likewise, PA was higher in boys than girls and decreased with age. The decline of PA during adolescence is a consistent finding in studies using self-report instruments [37] and has also been found using accelerometers [31].

Several limitations are present in this study. Firstly, there is no consensus on the most suitable accelerometer cut points to use for classifying MVPA in children or adolescents [41]. This study used Freedson cut points with moderate intensity ≥4METs. However, correlations between self-report and accelerometer data are similar for MVPA derived from cut points and the CPM obtained from raw data (Table 3), and the correlation strength would be described in the same way. Secondly, lack of compliance with wearing the accelerometer meant relatively high numbers were excluded from the analysis (56.1 % compliance for ≥5 days and 18.0 % compliance for seven days). A small sample size within certain groups limited the ability to draw definitive conclusions (e.g. male and primary students (*n* = 28) when using the seven-day criterion). Nonetheless, the final sample size was similar to previous validation studies [10, 12, 17]. Third, the attainment of the PAGL was quite low across the study. This influenced the estimation of sensitivity and PPV.

## Conclusions

Assessing non-compliance with PAGL is central to public health as it identifies the segment of the population that would benefit from increased PA. The PACE+ questionnaire was developed to identify youth not meeting PA recommendations. This study confirms the validity of the instrument for this purpose. Notably, validity is higher in females and older children. However, a series of different questionnaires for specific sex or age groups should be avoided, as the interpretation of youth population PA would be compromised. The ease of administration that this tool offers is vital for use at a population level [18]. The continued use of the questionnaire is recommended and will allow for comparability between studies, tracking of PA over time including trends in youth population prevalence. For more detailed information, it should be used alongside other measures that describe PA behaviour, e.g. measures for assessing specific types of physical activities.

## Abbreviations

PA: Physical activity
PAGL: Physical activity guidelines
MVPA: Moderate to vigorous physical activity
ICC: Intraclass correlation coefficient
CPM: Counts per minute
AM: Average method
ADM: All days method
PPV: Positive predictive value
NPV: Negative predictive value
